# Zhankuic Acids A, B and C from *Taiwanofungus camphoratus* Act as Cytotoxicity Enhancers by Regulating P-Glycoprotein in Multi-Drug Resistant Cancer Cells

**DOI:** 10.3390/biom9120759

**Published:** 2019-11-21

**Authors:** Yu-Ning Teng, Yen-Hsiang Wang, Tian-Shung Wu, Hsin-Yi Hung, Chin-Chuan Hung

**Affiliations:** 1 Department of Medicine, College of Medicine, I-Shou University, 8 Yida Road, Kaohsiung 82445, Taiwan; eunicegh520@gmail.com; 2 Department of Pharmacy, Nantou Hospital, Ministry of Health and Welfare, 478 Fuxing Rd., Nantou City, Nantou County 540, Taiwan; mon2328730@gmail.com; 3 Department of Pharmacy, College of Pharmacy, China Medical University, 91 Hsueh-Shih Road, Taichung 40402, Taiwan; 4 School of Pharmacy, College of Medicine, National Cheng Kung University, Tainan 701, Taiwan; tswu@mail.ncku.edu.tw (T.-S.W.); z10308005@email.ncku.edu.tw (H.-Y.H.); 5 Department of Pharmacy, College of Pharmacy and Health Care, Tajen University, Pingtung 907, Taiwan; 6 Department of Pharmacy, China Medical University Hospital, 2 Yude Road, Taichung 40447, Taiwan

**Keywords:** multidrug resistance, p-glycoprotein, *Taiwanofungus camphoratus*, Zhankuic acid

## Abstract

Since P-glycoprotein (P-gp)-related multidrug resistance (MDR) remains the most important unsolved problem in cancer treatment, scientists are attempting to find potential structures from natural resources. The aim of the present study was to elucidate whether the triterpenoids from *Taiwanofungus camphoratus* could reverse cancer MDR by influencing P-gp efflux pump. Substrates efflux assay and P-gp ATPase activity assay were conducted to reveal the molecular mechanisms of P-gp inhibition, while SRB assay, cell cycle analyses and apoptosis analyses were performed to confirm the cancer MDR modulating effects. The results indicated that Zhankuic acids A, B and C (ZA-A, ZA-B and ZA-C) impacted P-gp efflux function in competitive, noncompetitive and competitive manners, respectively. Furthermore, these triterpenoids all demonstrated inhibitory patterns on both basal P-gp ATPase activity and verapamil-stimulated ATPase activity. In terms of MDR reversal effects, ZA-A sensitized the P-gp over-expressing cell line (ABCB1/Flp-In^TM^-293) and MDR cancer cell line (KB/VIN) toward clinically used chemotherapeutic drugs, including doxorubicin, paclitaxel and vincristine, exhibiting the best cytotoxicity enhancing ability among investigated triterpenoids. The present study demonstrated that ZA-A, ZA-B and ZA-C, popular triterpenoids from *T. camphoratus*, effectively modulated the drug efflux transporter P-gp and reversed the cancer MDR issue.

## 1. Introduction

Despite ever-advancing science and technology in medical treatment, the cancer mortality rate remains the highest among numerous human diseases. Although several interventions are available for cancer treatment, such as surgery, radiation and immunotherapy, chemotherapy remains the most widely used treatment for overcoming malignancy [1]. Combinatorial chemotherapy addresses the resistance caused by single drug treatment; however, the ensuing side effects and multidrug resistance (MDR) can worsen the situation [1]. Several molecular mechanisms of resistance have been identified, including interrupted cellular apoptotic pathways, enhanced DNA damage repair and insufficient chemotherapeutic drug concentrations in cancer cells caused by reduced drug intake or increased drug efflux [2]. In cancer MDR, the overexpression of drug efflux transporter P-glycoprotein (P-gp) has been a major concern over the past 40 years [3]. P-gp is encoded by ABCB1, which belongs to the adenosine triphosphate-binding cassette superfamily. P-gp can recognize and pump out numerous hydrophobic drugs, including chemotherapeutic agents, by changing its conformation under ATP consumption [4].

To overcome P-gp-related MDR, several strategies such as targeting, evading and inhibiting P-gp have been tested. However, off-target effects and the ability of P-gp to recognize diverse structures have limited the application of several methods [2]. In reviewing the history of P-gp inhibitor development, the major reasons of failure include affinity, specificity and safety. First- and second-generation inhibitors were clinically limited because their substrate characteristics for P-gp and cytochrome P450, respectively, which resulted in complex drug–drug interactions and higher dose requirements [5,6]. Although third-generation inhibitors were developed using the quantitative structure-activity relationship for developing more potent and selective agents, the severe side effects caused by these chemically synthesized drugs still impede their clinical application [7]. Therefore, the development of fourth-generation P-gp inhibitors focuses on safety. Compounds or their derivatives from natural products are receiving attention because of their higher safety index than that of chemically sourced agents [8].

Resources are abundant in nature, including in plants, fungi and marine organisms. Curcuminoids are well-known P-gp inhibitors from plants, and nanotechnological pharmaceutical development and chemical modification are underway to improve the effects of these promising candidates [9]. Regarding fungi, a study demonstrated that crude ethanol extracts from Antrodia cinnamomea fruiting bodies are potent P-gp inhibitors [10]. Several index components in *Taiwanofungus camphoratus* (A. cinnamomea), such as adenosine, cordycepin and Zhankuic acid compounds, are ergostane-type triterpenoids [11]. Zhankuic acids A (ZA-A), B (ZA-B) and C (ZA-C) are structurally related compounds that were successfully separated in 1995 [12]. ZA-A and ZA-C have anti-inflammatory and cytotoxic activity, whereas ZA-B exhibits weak anticholinergic and antiserotonergic effects [11,13]. ZA-A and ZA-C exhibited cytotoxic abilities in mouse leukemia cell line P-388 with an IC_50_ of 1.8 and 5.4 μg/mL, respectively. Another study revealed that ZA-A and ZA-C could induce cell apoptosis in colon cancer cell lines HT-29 and SW-480 [14,15]. Nevertheless, the P-gp inhibitory and cancer MDR reversal effects of these triterpenoids remain unclear and warrant further investigation.

In our study, ZA-A, ZA-B and ZA-C were derived from *T. camphoratus* to research their inhibitory effects and mechanisms on human drug efflux transporter P-gp. The cancer MDR-reversing ability and underlying cytotoxic mechanisms of these triterpenoids were also elucidated. ZA-A, ZA-B and ZA-C, the popular triterpenoids from *T. camphoratus*, effectively modulated the drug efflux transporter P-gp and reversed cancer MDR.

## 2. Materials and Methods

### 2.1. Chemicals and Reagents

Dulbecco’s Modified Eagle Medium, RPMI 1640 medium, fetal bovine serum (FBS), phosphate buffered saline (PBS; pH 7.2), trypsin-EDTA and hygromycin B were purchased from Invitrogen (Carlsbad, CA, USA). Zeocin was from InvivoGen (San Diego, CA, USA). Acetic acid, β-Mercaptoethanol (β-ME), dimethyl sulfoxide (DMSO), ethanol (absolute; analytical grade), paclitaxel, rhodamine 123, sulforhodamine B (SRB), trichloroacetic acid (TCA), Tris Base, (±)-verapamil and vincristine were obtained from Sigma-Aldrich Co. (St. Louis, MO, USA). Calcein-AM was from AAT Bioquest (Sunnyvale, CA, USA), and doxorubicin was from US Biological (Woburn, MA, USA). ZA-A, ZA-B and ZA-C were extracted and separated from fruiting body of *T. camphoratus* by Dr. Tian-Shung Wu’s laboratory (National Cheng-Kung University, Tainan, Taiwan) [11,16].

### 2.2. Cell Lines

The human P-gp stable expression cells (ABCB1/Flp-In^TM^-293) and parental cell line Flp-In^TM^-293 were constructed as previous described [17]. Human cervical epithelioid carcinoma HeLaS3 was purchased from Bioresource Collection and Research Center (Hsinchu, Taiwan). The multi-drug-resistant human cervical cancer cell line KB/VIN was a generous gift from Dr. Kuo-Hsiung Lee (University of North Carolina, Chapel Hill, NC, USA). The resistance of KB/VIN was maintained with regular vincristine treatment. All cells were cultured in DMEM or RPMI-1640 containing 10% FBS at 37 °C in a humidified atmosphere of 5% CO_2_.

### 2.3. SRB Cytotoxicity Assay

Briefly, after 72 h treatment of series concentrations of chemotherapeutic drugs with or without ZA-A, ZA-B or ZA-C, 50% trichloroacetic acid (TCA) was added to fix cells for 30 min, and then the cells were washed with water and air-dried. Next day, cells were then stained with 0.04% sulforhodamine B (SRB) for 30 min, and then the unbound dye was removed by washing the cells with 1% acetic acid. Next day, the bound stain was solubilized with 10 mM Tris Base before absorbance detection. The absorbance was measured at 515 nm using a BioTek Synergy HT Multi-Mode Microplate Reader (BioTek Instruments, Inc., Winooski, VT, USA).

### 2.4. Intracellular Calcein Accumulation Assay

The method has been described in our previous research [9]. The calcein fluorescence generated within the cells was detected by BioTek Synergy HT Multi-Mode Microplate Reader using excitation wavelength 485 nm and emission wavelength 528 nm at 37 °C temperature every 3 min for 30 min.

### 2.5. Real-Time Quantitative RT-PCR

The method has been described in our previous research [9]. The relative ABCB1 mRNA expression levels were normalized to the amount of GAPDH in the same cDNA and evaluated by StepOnePlus^TM^ Real-Time PCR System (Applied Biosystems^®^, Waltham, MA, USA).

### 2.6. MDR1 Shift Assay

The method has been described in our previous research [9]. The conformation change of P-gp after the addition of ZA-A, ZA-B or ZA-C was examined by using a MDR1 Shift Assay kit (EMD Millipore Corp., Billerica, MA, USA) according to the manufacturer’s protocol. The fluorescence was measured by FACS analysis (BD FACSCanto System).

### 2.7. Rhodamine123 and Doxorubicin Efflux Assay

The method has been described in our previous research [9]. The fluorescence of rhodamine123 and doxorubicin was measured using a BioTek Synergy HT Multi-Mode Microplate Reader (excitation/emission: 485/528 nm for rhodamine123, 485/590 nm for doxorubicin). Scientist v2.01 (MicroMath Scientific Software, Salt Lake City, UT, USA) was used to estimate the kinetic parameters by nonlinear regression according to the following equation (1):

V = (V_max_ × C)/(K_m_ + C) (1)

where V denotes the efflux rate; V_max_, the maximal efflux rate; K_m_, the Michaelis-Menten constant; and C, the substrate concentration.

### 2.8. P-gp ATPase Activity Assay

The method has been described in our previous research [9]. For the evaluation of P-gp ATPase activity of ZA-A, ZA-B and ZA-C, Pgp-Glo^TM^ Assay System from Promega (Madison, WI, USA) was used. Luminescence was measured using a BioTek Synergy HT Multi-Mode Microplate Reader, and data were presented as change in luminescence (ΔRLU).

### 2.9. Cell Cycle Analysis

Cells were plated to 6-well plates with serum-free medium for starvation. 24 h later, cells were treated with chemotherapeutic drugs with or without ZA-A, ZA-B or ZA-C for 72 h. After that, cells were harvested and washed in cold phosphate-buffered saline (PBS) and then fixing in ice-cold 70% ethanol overnight. Next day, cells were incubated with 50 μg/mL PI at 4 °C overnight. Next day, the fluorescence of cells was analyzed by FACS analysis (BD FACSCanto System with excitation laser 488 nm, measuring at emission 575 nm for PI).

### 2.10. Apoptosis Assay

Apoptosis evaluation was performed with Alexa Fluor^®^ 488 annexin V/Dead Cell Apoptosis Kit from MOLECULAR PROBES^®^ (Cat. No. V13241, Eugene, OR, USA). Cells were plated to 6-well plates and incubated overnight. On the next day, cells were treated with chemotherapeutic drugs with or without ZA-A, ZA-B or ZA-C for 72 h. After that, cells were harvested and washed in cold phosphate-buffered saline (PBS) and then re-suspended in 1× annexin-binding buffer. 5 μL Alexa Fluor^®^ 488 annexin V and 1 μL 100 μg/mL PI working solution were added to each 100 μL cell suspension. The cells were incubated at room temperature for 15 min. After that, 400 μL 1× annexin-binding buffer were added to each sample. The fluorescence of the stained cells was then analyzed by FACS analysis (BD FACSCanto System (Becton, Dickinson and Company, San Jose, CA, USA) with excitation laser 488 nm, measuring at emission 530 nm for FITC and 575 nm for PI, respectively).

### 2.11. Statistical Analysis

ANOVA followed by post hoc analysis (Tukey’s test) or student’s *t*-test were used to evaluate statistical differences. The statistical significance was set at *p* < 0.05.

## 3. Results

### 3.1. P-gp Efflux Function was Inhibited by ZA-A, ZA-B and ZA-C

Before conducting P-gp efflux function experiments, the cytotoxicity of ZA-A, ZA-B and ZA-C was examined by SRB assay to choose a safer drug concentration range for the following functional tests. As Figure S1a–d showed, the IC_50_ of ZA-A, ZA-B and ZA-C were all larger than 40 μM in Flp-In^TM^-293, ABCB1/Flp-In^TM^-293 (over-expressing P-gp), HeLaS3 and KB/VIN (multi-drug resistant cancer cells, P-gp overexpression).

**Figure 1:**
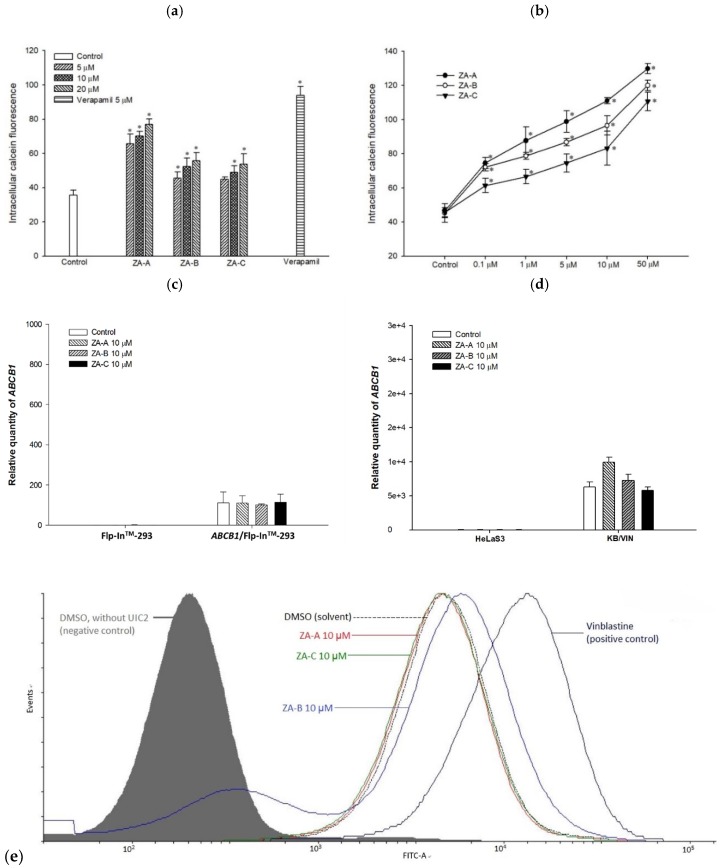
The effects of Zhankuic acids A, B and C (ZA-A, ZA-B and ZA-C) on human P-glycoprotein (P-gp). (**a**) Intracellular calcein fluorescence with or without ZA-A, ZA-B or ZA-C pretreatment in ABCB1/Flp-In^TM^-293 cell line (over-expressing human P-gp). Verapamil 5 μM was used as a positive control. * denotes *p* < 0.05 compared with the intracellular calcein fluorescence in control group. (**b**) The dose-dependent effects of ZA-A, ZA-B or ZA-C on calcein retention in ABCB1/Flp-In^TM^-293. * denotes *p* < 0.05 compared with the intracellular calcein fluorescence in control group. (**c**), (**d**) There were no significant ABCB1 mRNA expression differences after treating the ABCB1/Flp-In^TM^-293 or KB/VIN with 10 μM ZA-A, ZA-B or ZA-C for 72 h. © The conformation of P-gp was not influenced under the treatment of 10 μM ZA-A, ZA-B or ZA-C. Vinblastine was used as a positive control.

The primary screening results of P-gp inhibitory effects are exhibited in Figure 1a; ZA-A, ZA-B and ZA-C all increased intracellular calcein retention levels higher than the positive control verapamil did, indicating P-gp efflux function was prohibited in ABCB1/Flp-In^TM^-293. Further dose-response study also revealed that these triterpenoids significantly inhibit ABCB1/Flp-In^TM^-293 cell P-gp efflux behavior in a dose-dependent manner, with ZA-A giving the best modulating effect (Figure 1b).

### 3.2. ZA-A, ZA-B and ZA-C were not P-gp Substrates and the ABCB1 mRNA Levels were not Influenced by These Triterpenoids

In order to understand whether the ABCB1 mRNA levels were affected by ZA-A, ZA-B and ZA-C in the long-term treatment, real-time quantitative RT-PCR was performed. Although P-gp’s efflux function was inhibited, the ABCB1 mRNA levels were not significantly influenced by these triterpenoids under 72 h treatment, unlike in P-gp over-expressing cell line (ABCB1/Flp-In^TM^-293) or MDR cancer cell line (KB/VIN) (Figure 1c,d).

The other concern was whether ZA-A, ZA-B and ZA-C were P-gp’s substrates. As MDR1 shift assay revealed, the conformational change of P-gp was not obvious under the treatment of all three triterpenoids, and the fluorescence peaks did not shift in right direction as the positive control vinblastine (A standard P-gp substrate) did in ABCB1/Flp-In^TM^-293, indicating ZA-A, ZA-B and ZA-C were not substrates of P-gp (Figure 1e).

### 3.3. The Inhibitory Mechanisms and ATPase Interactions between P-gp and ZA-A, ZA-B and ZA-C

**Figure 2:**
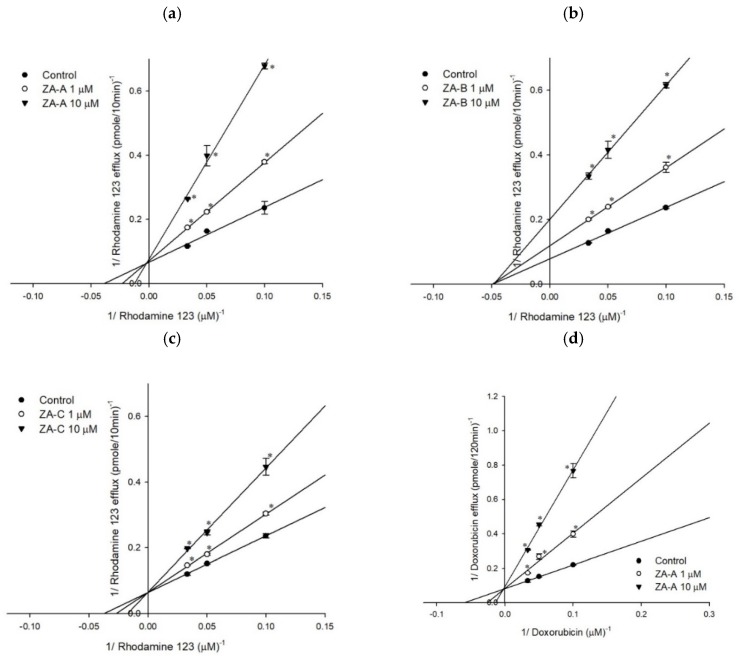

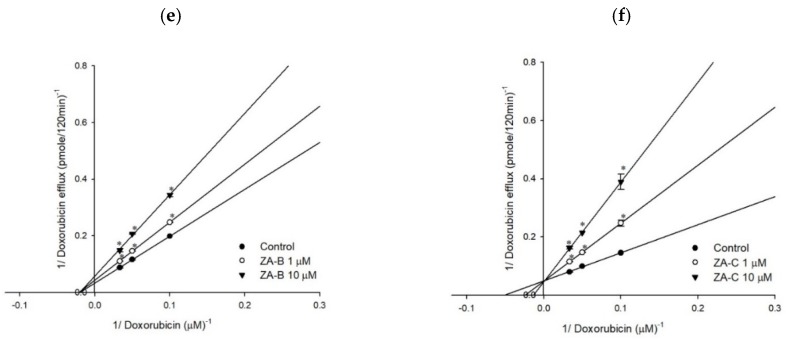
The Lineweaver–Burk plot analyses of ZA-A, ZA-B and ZA-C on the transport of rhodamine123 or doxorubicin in human P-gp. (**a**) ZA-A, (**b**) ZA-B and (**c**) ZA-C inhibited rhodamine 123 efflux via competitive, noncompetitive, and competitive mechanisms, respectively. (**d**) ZA-A, (**e**) ZA-B and (**f**) ZA-C inhibited doxorubicin efflux via competitive, noncompetitive and competitive mechanisms, respectively. * *p* < 0.05 compared with rhodamine123 or doxorubicin efflux without ZA-A, ZA-B or ZA-C treatment (control group). Data were presented as mean ± SE of at least three experiments, each in triplicate.

The P-gp inhibitory mechanisms were evaluated by rhodamine123 and doxorubicin efflux assay. As Tables S1 and S2 revealed, increased ZA-A and ZA-C concentrations led to elevated K_m_ value and constant V_max_ value, regardless of whether the fluorescent substrate was rhodamine123 or doxorubicin, indicating the inhibitory kinetic mechanism was competitive inhibition (Figure 2a,c,d,f). On the other hand, ZA-B showed noncompetitive inhibition with both P-gp substrates rhodamine123 and doxorubicin in the Lineweaver-Burk plot analyses, affecting V_max_ value but not K_m_ value in the inhibitory kinetic studies (Figure 2b,e; Tables S1 and S2).

**Figure 3:**
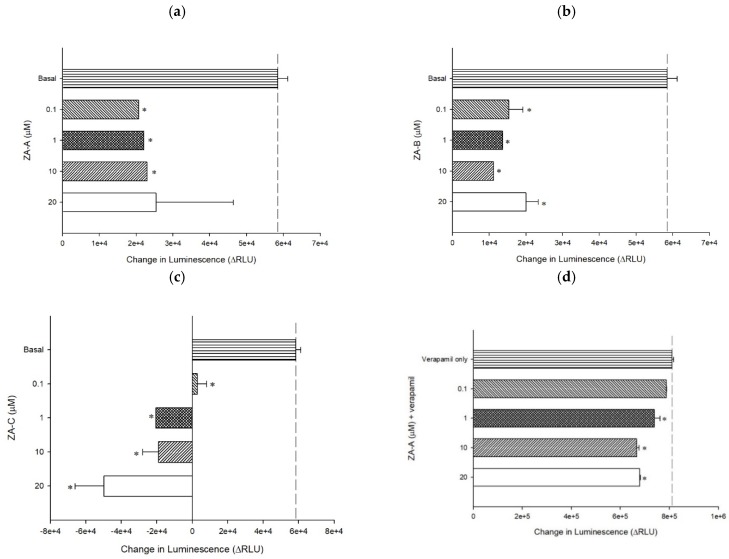

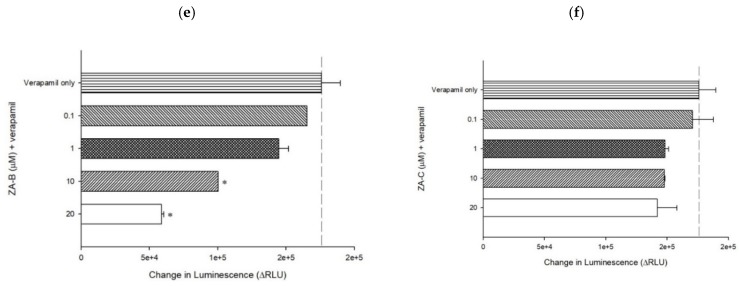
The P-gp ATPase modulating effects of ZA-A, ZA-B and ZA-C. (**a**) ZA-A, (**b**) ZA-B and (**c**) ZA-C inhibited basal ATPase activity in a dose-dependent trend. Verapamil-stimulated ATPase activity was reversed by (**d**) ZA-A, (**e**) ZA-B and (**f**) ZA-C. Data were analyzed in terms of the change of luminescence (ΔRLU). * denotes *p* < 0.05 compared with theΔRLU in basal activity group or 200 μM verapamil treatment group. Data were presented as mean ± SE of at least three experiments, each in triplicate.

The influence of ZA-A, ZA-B and ZA-C on P-gp ATPase energy consumption was investigated by ATPase assay. The results revealed that all three triterpenoids significantly inhibited basal ATPase activity, resulting in decreased energy production on ATPase site for efflux function (Figure 3a–c). When combined with verapamil, ZA-A, ZA-B and ZA-C also showed inhibitory trend on verapamil-stimulated ATPase activity, indicating their negative regulating ability on ATPase function (Figure 3d–f).

### 3.4. The MDR Reversal Effects and Molecular Mechanisms of ZA-A, ZA-B and ZA-C

To examine whether the P-gp inhibitory effects could be translated to MDR reversal ability, the combinatory cytotoxicity of these triterpenoids and chemotherapeutic drugs was performed. As Table 1 and Table 2 show, the ABCB1/Flp-In^TM^-293 and KB/VIN had significant resistance to chemotherapeutic drugs, compared to their parental cells, Flp-In^TM^-293 and HeLaS3, respectively. The IC_50_ of doxorubicin, paclitaxel, and vincristine decreased with the combination of ZA-A, ZA-B and ZA-C in both P-gp over-expressing cell line (ABCB1/Flp-In^TM^-293) and MDR cancer cell line (KB/VIN). The reversal effect of ZA-A was the most prominent; when treating with 20 μM in KB/VIN, the reversal folds were 50.0, 11.1 and 51.7 for doxorubicin, paclitaxel and vincristine, respectively.

**Figure 4:**
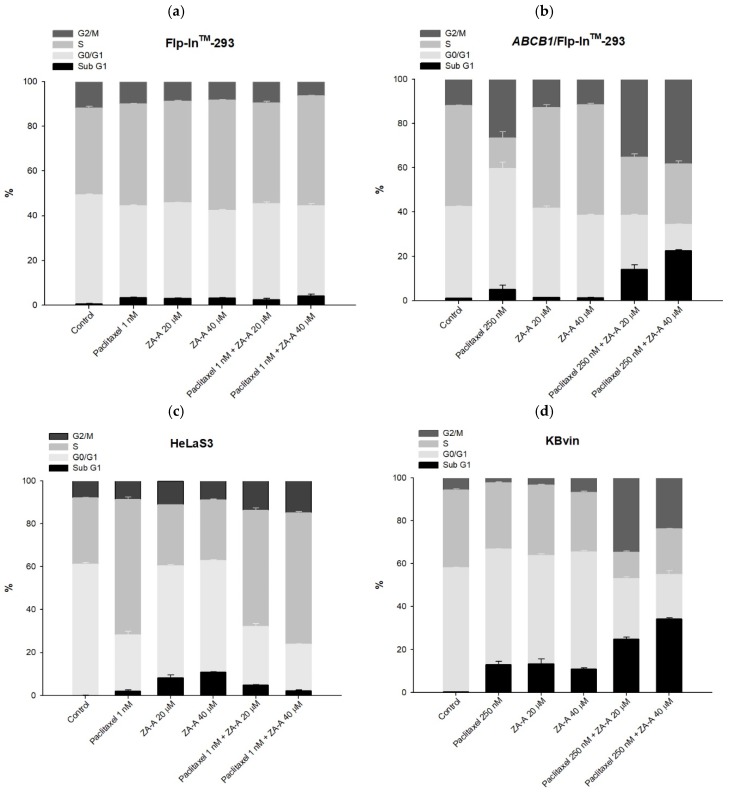

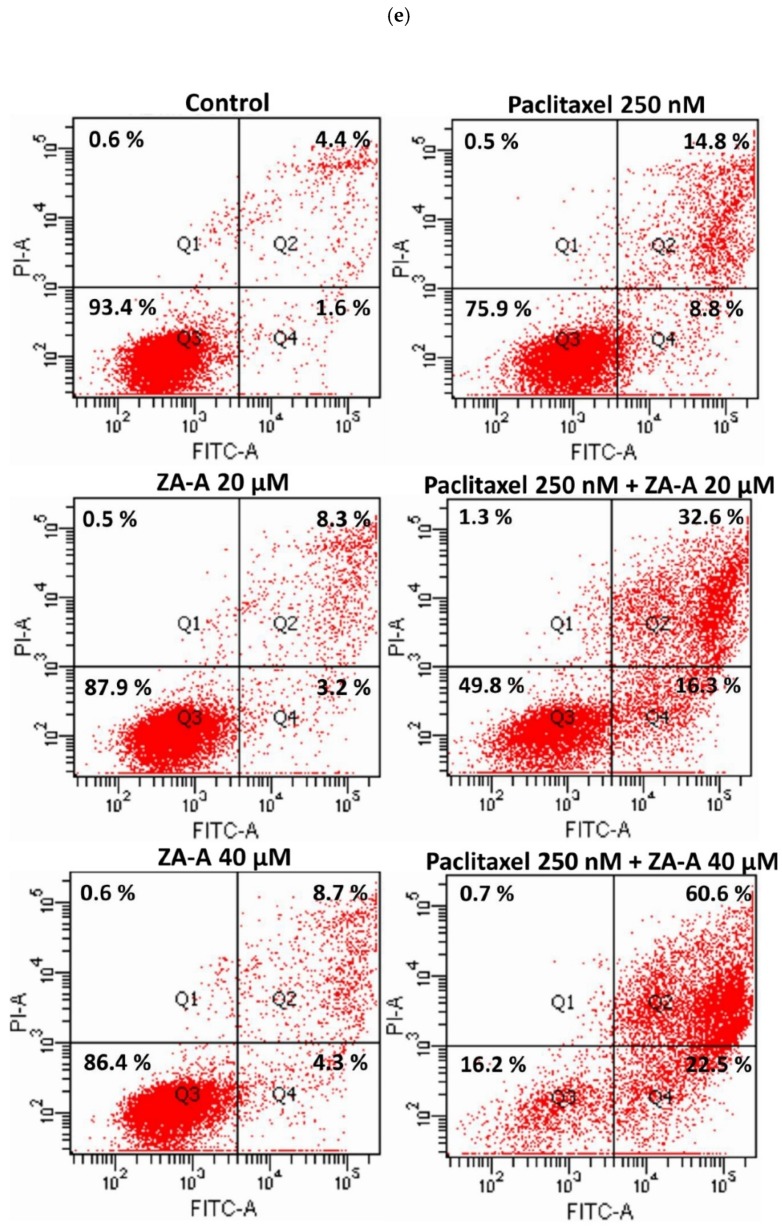
The cell cycle and apoptosis analyses of ZA-A and paclitaxel combinatorial treatment. (**a**), (**b**), (**c**) and (**d**) The cell cycle distribution of 72 h treatment in Flp-In^TM^-293, ABCB1/Flp-In^TM^-293, HeLaS3 and KB/VIN, respectively. Data were presented as mean ± SE of at least three experiments, each in triplicate. (**e**) The apoptosis phenomenon of 72 h treatment in KB/VIN cell line. Apoptosis and necrosis status of each sample was determined by annexin V (X-axis FITC) and PI (Y-axis PI). Cells distributed in Q1, Q2, Q3 and Q4 represent necrosis, late-apoptosis and normal and early-apoptosis, respectively.

The following MDR reversal molecular mechanisms were further investigated with ZA-A, which exhibited the strongest potency in the cytotoxicity enhancement. In the cell cycle analysis, compared to the paclitaxel 250 nM group, the combination of paclitaxel and ZA-A led to increased G2/M and sub G1 percentage in P-gp over-expressing cell line (ABCB1/Flp-In^TM^-293), while keeping cell cycle distribution constant in parental cell line Flp-In^TM^-293 (Figure 4a,b, Table S3, Figure S2a,b). The same modulating pattern could also be observed in HeLaS3 and KB/VIN. In the MDR cancer cell KB/VIN, adding ZA-A to paclitaxel treatment resulted in G2/M arrest and sub G1 accumulation, indicating the resistant phenomenon was successfully reversed (Figure 4c,d, Table S3, Figure S2c,d).

The enhanced cell apoptotic effects by ZA-A were further approved by apoptosis assay. Compared to parental cells (Flp-In^TM^-293 and HeLaS3), the percentage of early-apoptotic and late-apoptotic cells largely increased with the add-on of ZA-A to paclitaxel in resistant cells (ABCB1/Flp-In^TM^-293 and KB/VIN) (Figure S3a–c, Figure 4e). In KB/VIN, the early-apoptotic region increased from 8.8% (paclitaxel 250 nm) to 16.3% and 22.5% when combined with ZA-A 20 μM and 40 μM, respectively (Figure 4e). Above molecular investigation results demonstrated that ZA-A significantly reversed the paclitaxel resistance and enhanced cytotoxicity by cell cycle G2/M arrest and pro-apoptotic effects.

## 4. Discussion

The investigation of natural products on the application of MDR cancer treatment is receiving growing attention. The present study demonstrated that ZA-A, ZA-B and ZA-C, popular triterpenoids from *T. camphoratus*, could reverse difficult-treating multi-drug resistance by functionally modulating drug efflux transporter P-gp. These triterpenoids were not substrates of P-gp, and their gene expression was not altered in the long period of treatment. ZA-A and ZA-C impacted P-gp via competitive inhibition, while ZA-B inhibited P-gp in a noncompetitive manner. Furthermore, these triterpenoids all demonstrated inhibitory patterns not only in basal P-gp ATPase activity but also in verapamil-stimulated ATPase activity. In terms of MDR reversal effects, ZA-A exhibited the best cytotoxicity-enhancing ability, sensitizing the P-gp over-expressing cell line (ABCB1/Flp-In^TM^-293) and MDR cancer cell line (KB/VIN) toward several chemotherapeutic drugs, such as doxorubicin, paclitaxel and vincristine. Further study revealed that combining ZA-A and paclitaxel significantly elevated the G2/M arrest and sub G1 accumulation levels, rendering the resistant cell line’s cell apoptosis and death.

With regard to whether the P-gp gene expression level was influenced by natural triterpenoids, previous studies showed controversial results. Toosendanin down-regulated ABCB1 mRNA expression level under 48 h treatment in resistant breast cancer cell MCF-7/ADM [18], whereas sipholane A 72 h treatment exhibited no influence on P-gp expression level in drug-resistant human colon cancer cell line SW620/Ad300 [19]. Besides, acerinol also demonstrated no impact on the ABCB1 mRNA level in both HepG2/ADM and MCF-7/ADR multi-drug resistant cancer cells [20]. Above discrepancies can be attributed to the diverse structures among natural triterpenoids that exhibited different side chain elongation or cyclization linkage. Previous studies also revealed that crude extracts from *T. camphoratus* showed inconsistent results on the P-gp gene expression regulation, depending on whether the extracts were from fruiting body or mycelium [21,22]. In our previous research, we demonstrated that ethanol extracts from *T. camphoratus* (*Antrodia cinnamomea*) fruiting body had no influence on ABCB1 mRNA level under 24, 48 and 72 h treatment [10]. In the present study, pure triterpenoids compounds, ZA-A, ZA-B and ZA-C from *T. camphoratus*, exhibited constant ABCB1 mRNA quantities in both ABCB1/Flp-In^TM^-293 and KB/VIN cell lines after 72 h treatment.

One reason for the clinical failures of previous P-gp inhibitors was that the inhibitors themselves were also substrates of P-gp; therefore, higher drug concentrations were needed to achieve P-gp modulating effects, followed by ensuing off-target toxicity. The present drug development aims at discovering novel agents that are not substrates of P-gp to avoid previous negative experiences. In our previous research, higher concentrations (10 μg/mL and 20 μg/mL) of ethanol extracts from *T. camphoratus* fruiting body were demonstrated P-gp’s substrate through performing MDR1 shift assay [10]. However, in the present study, ZA-A, ZA-B and ZA-C were experimentally proven to not influence the conformational change of P-gp, meaning they were not substrates of P-gp. These discrepant results could be due to the multi-components in the ethanol extracts; there are not only triterpenoids in the crude extracts, but other structure-unrelated natural compounds like adenosine and cordycepin.

In terms of the inhibitor binding sites on the P-gp and its ATPase, detailed information could be revealed by rhodamine123 and doxorubicin efflux assay, as well as ATPase activity assay. Previous studies revealed that there were at least three drug binding sites on P-gp [23], for example, the fluorescent substrate rhodamine123 bond to R and M sites, while chemotherapeutic drug doxorubicin attached to R site. Hence, based on our present results, we could interpret that ZA-A and ZA-C competed the R site with rhodamine 123 and doxorubicin, and the M site with rhodamine123. ZA-B showed no competition at the R and M site, implying there was allosteric P-gp regulation mechanism. However, the complexity of drug binding at P-gp still warrants further investigation to ascertain the binding modes. The other kind of binding happens at P-gp ATPase binding site, where the consumption of ATP regulates the drug efflux behavior of P-gp. According to the classification rules from previous researches [24], ZA-A, ZA-B and ZA-C could be categorized to Class III P-gp inhibitors, as they inhibited not only basal ATPase activity but verapamil-stimulated ATPase activity. Furthermore, the activity stimulated by standard stimulator verapamil was diminished by the addition of Zhankuic acid compounds, implying that ZA-A, ZA-B and ZA-C would compete the ATPase binding site with verapamil in a dose-dependent pattern.

The MDR reversing effects of ZA-A, ZA-B and ZA-C were related to P-gp inhibitory ability in the present study. With the co-treatment of Zhankuic acid compounds, the intracellular concentrations of chemotherapeutic drugs were increased by blocking the P-gp pump out system. Compared to the resistant cases in which almost all chemotherapeutic drugs were pumped out, higher exposure of cytotoxic agents rendered cell apoptosis and death in MDR cancer cell line. Reviewing past related research, other triterpenoids could also enhance the cytotoxicity of chemotherapeutic drugs. For instance, when combining ethyllucidenates A and vincristine in the treatment of CML cancer cell line K562/A02 that over-expressed P-gp, the percentage of G2/M arrest was significantly larger than the vincristine only group [25]. The early-apoptotic phenomenon of MCF-7/ADR was promoted by the combination of pristimerin and doxorubicin [26]. Ginsenoside Rg3 enhanced the intrinsic cell apoptosis pathway of cyclophosphamide to delay the tumor growth in hepatocellular cancer cell line Hep1-6 inoculated mice and prolong their lifespan [27,28]. Herein, ZA-A helped paclitaxel arrest KB/VIN at cell cycle G2/M phase and promote apoptosis; however, whether the apoptosis is related to intrinsic or extrinsic apoptotic pathways still needs further investigation.

In addition, our results showed that the combination of Zhankuic acid compounds with chemotherapeutic drugs could also enhance the cytotoxicity in drug-sensitive cell line HeLaS3, indicating there are other molecular mechanisms for cytotoxicity. ZA-A was reported to have anti-inflammatory functions such as the inhibition of ROS produced by fMLF- or PMA-activated peripheral human neutrophils [29], or modulating the production of NO, iNOS, COX2, TNF-α and IL-6, in macrophages [30]. The relationship between anti-inflammation and the cytotoxic effects of ZA-A deserves further experiment investigation to understand the diverse pharmacological roles of ZA-A. In order to avoid the potential drug–drug interaction effect in vivo, some pharmaceutical modification could be adopted. For instance, these compounds could be wrapped in advance and labeled with antibody. By creating physical barriers, the potential drug–drug interaction effect in vivo might be reduced.

**Figure 5:**
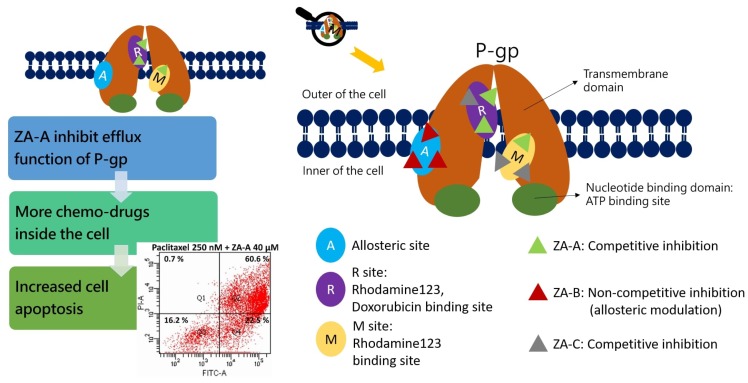
Summary of the P-gp inhibitory mechanisms and multidrug resistance (MDR) reversing effects of Zhankuic acids. ZA-A, ZA-B and ZA-C impacted P-gp efflux function through competitive, noncompetitive and competitive inhibitions, respectively. These effects resulted in increased intracellular concentration of chemo-drugs, rendering cell apoptosis.

Taking the above descriptions together, the present study demonstrated the P-gp inhibitory and MDR reversal ability of ZA-A, ZA-B and ZA-C. The summarized effects and mechanisms of Zhankuic acids are exhibited in Figure 5. The future application of these triterpenoids on the multi-drug resistant cancer treatment was promising. Considering the bitter taste of triterpenoids, the pharmaceutical design could use sugar coating or flavoring agents to improve the taste perception.

## Figures and Tables

**Table 1:** The reversal effects of ZA-A, ZA-B and ZA-C on chemotherapeutic drug resistance in P-gp over-expressing cell line ABCB1/Flp-In^TM^293.

**Cell Line**	**Flp-In^TM^293**		**ABCB1/Flp-In^TM^293**	
**Compound**	**IC_50_ ± S.E. (nM)**	**RF**	**IC_50_ ± S.E. (nM)**	**RF**
**Doxorubicin**	84.783 ± 0.856	1.0	767.708 ± 135.973	1.0
+ ZA-A (10 μM)	53.276 ± 1.913	1.6	462.964 ± 27.210	1.7
+ ZA-A (20 μM)	44.824 ± 0.436	1.9	433.570 ± 9.658	1.8
+ ZA-A (40 μM)	37.224 ± 2.369 *	2.3	124.221 ± 14.013 *	6.2
+ ZA-B (10 μM)	50.806 ± 7.431	1.7	462.296 ± 39.402	1.7
+ ZA-B (20 μM)	48.319 ± 6.729	1.8	414.329 ± 18.101	1.9
+ ZA-B (40 μM)	42.509 ± 2.711 *	2.0	208.154 ± 3.263 *	3.7
+ ZA-C (10 μM)	64.002 ± 1.282	1.3	555.031 ± 6.192	1.4
+ ZA-C (20 μM)	56.275 ± 2.034	1.5	507.168 ± 26.490	1.5
+ ZA-C (40 μM)	45.570 ± 0.215	1.9	215.486 ± 105.299 *	3.6
**Cell Line**	**Flp-In^TM^293**		**ABCB1/Flp-In^TM^293**	
**Compound**	**IC_50_ ± S.E. (nM)**	**RF**	**IC_50_ ± S.E. (nM)**	**RF**
**Paclitaxel**	6.556 ± 0.037	1.0	381.099 ± 19.440	1.0
+ ZA-A (10 μM)	6.342 ± 0.336	1.0	119.886 ± 1.945 *	3.2
+ ZA-A (20 μM)	4.649 ± 0.314	1.4	33.885 ± 7.803 *	11.2
+ ZA-A (40 μM)	1.607 ± 0.638 *	4.1	4.099 ± 1.729 *	93.0
+ ZA-B (10 μM)	5.749 ± 0.021	1.1	243.946 ± 26.032	1.6
+ ZA-B (20 μM)	4.586 ± 0.182	1.4	172.364 ± 9.353 *	2.2
+ ZA-B (40 μM)	4.362 ± 0.650	1.5	27.096 ± 2.799 *	14.1
+ ZA-C (10 μM)	5.089 ± 0.752	1.3	312.457 ± 3.101	1.2
+ ZA-C (20 μM)	4.534 ± 0.063	1.4	285.500 ± 3.030	1.3
+ ZA-C (40 μM)	3.844 ± 0.297	1.7	201.582 ± 8.884	1.9
**Cell Line**	**Flp-In^TM^293**		**ABCB1/Flp-In^TM^293**	
**Compound**	**IC_50_ ± S.E. (nM)**	**RF**	**IC_50_ ± S.E. (nM)**	**RF**
**Vincristine**	83.822 ± 1.350	1.0	3858.781 ±217.537	1.0
+ ZA-A (10 μM)	85.570 ± 0.120	1.0	1868.840 ± 12.138 *	2.1
+ ZA-A (20 μM)	76.274 ± 9.852	1.1	1200.585 ± 112.243 *	3.2
+ ZA-A (40 μM)	3.732 ± 0.040 *	22.5	134.419 ± 4.400 *	28.7
+ ZA-B (10 μM)	43.234 ± 1.928	1.9	1698.434 ± 325.352 *	2.3
+ ZA-B (20 μM)	36.645 ± 12.895 *	2.3	1475.014 ± 111.837 *	2.6
+ ZA-B (40 μM)	4.459 ± 0.455 *	18.8	399.390 ± 30.490 *	9.7
+ ZA-C (10 μM)	69.245 ± 0.871	1.2	2995.200 ± 51.279	1.3
+ ZA-C (20 μM)	54.267 ± 1.594	1.5	2274.225 ± 75.201	1.7
+ ZA-C (40 μM)	7.801 ± 1.350 *	10.7	1553.927 ± 33.018 *	2.5

* *p* < 0.05 compared to chemotherapeutic drug treatment (doxorubicin, paclitaxel or vincristine) without ZA-A, ZA-B and ZA-C. The reversal fold (RF) was calculated by dividing the individual IC_50_ of chemotherapeutic drugs by the IC_50_ of chemotherapeutic drugs in the presence of ZA-A, ZA-B or ZA-C.

**Table 2:** The reversal effects of ZA-A, ZA-B and ZA-C on chemotherapeutic drug resistance in MDR cancer cell line KB/VIN.

**Cell Line**	**HeLaS3**		**KB/VIN**	
**Compound**	**IC_50_ ± S.E. (nM)**	**RF**	**IC_50_ ± S.E. (nM)**	**RF**
**Doxorubicin**	101.088 ± 1.283	1.0	3819.266 ± 98.110	1.0
+ ZA-A (10 μM)	75.675 ± 0.732	1.3	427.791 ± 49.019 *	8.9
+ ZA-A (20 μM)	51.914 ± 1.216	1.9	76.419 ± 0.151 *	50.0
+ ZA-B (10 μM)	105.041 ± 6.633	1.0	2109.439 ± 24.634	1.8
+ ZA-B (20 μM)	69.211 ± 5.204	1.5	1310.133 ± 1.797 *	2.9
+ ZA-C (10 μM)	99.561 ± 8.025	1.0	2146.430 ± 129.070	1.8
+ ZA-C (20 μM)	83.740 ± 2.555	1.2	621.683 ± 17.651 *	6.1
**Cell Line**	**HeLaS3**		**KB/VIN**	
**Compound**	**IC_50_ ± S.E. (nM)**	**RF**	**IC_50_ ± S.E. (nM)**	**RF**
**Paclitaxel**	4.994 ± 0.077	1.0	510.512 ± 11.020	1.0
+ ZA-A (10 μM)	1.509 ± 0.032 *	3.3	141.745 ± 0.293 *	3.6
+ ZA-A (20 μM)	0.793 ± 0.028 *	6.3	45.807 ± 0.128 *	11.1
+ ZA-B (10 μM)	2.053 ± 0.002 *	2.4	225.581 ± 1.992 *	2.3
+ ZA-B (20 μM)	0.609 ± 0.004 *	8.2	139.363 ± 2.287 *	3.7
+ ZA-C (10 μM)	3.657 ± 0.031	1.4	247.282 ± 7.276 *	2.1
+ ZA-C (20 μM)	3.238 ± 0.146	1.5	215.503 ± 5.563 *	2.4
**Cell Line**	**HeLaS3**		**KB/VIN**	
**Compound**	**IC_50_ ± S.E. (nM)**	**RF**	**IC_50_ ± S.E. (nM)**	**RF**
**Vincristine**	17.326 ± 0.645	1.0	15730.719 ± 2402.023	1.0
+ ZA-A (10 μM)	6.661 ± 0.820 *	2.6	2007.168 ± 51.760 *	7.8
+ ZA-A (20 μM)	4.463 ± 0.549 *	3.9	304.132 ± 28.753 *	51.7
+ ZA-B (10 μM)	8.838 ± 0.420 *	2.0	1967.489 ± 98.547 *	8.0
+ ZA-B (20 μM)	6.590 ± 0.236 *	2.6	1018.696 ± 138.704 *	15.4
+ ZA-C (10 μM)	17.004 ± 1.944	1.0	6952.094 ± 54.567 *	2.3
+ ZA-C (20 μM)	15.795 ± 0.928	1.1	2938.459 ± 874.146 *	5.4

* *p* < 0.05 compared to chemotherapeutic drug treatment (doxorubicin, paclitaxel or vincristine) without ZA-A, ZA-B and ZA-C. The reversal fold (RF) was calculated by dividing the individual IC_50_ of chemotherapeutic drugs by the IC_50_ of chemotherapeutic drugs in the presence of ZA-A, ZA-B or ZA-C.

## Supplementary Materials

The following are available online at https://www.mdpi.com/2218-273X/9/12/759/s1, Figure S1: The cytotoxicity of ZA-A, ZA-B and ZA-C in (a) Flp-In^TM^-293, (b) ABCB1/Flp-In^TM^-293, (c) HeLaS3 and (d) KB/VIN, respectively, Figure S2: The individual results of cell cycle distribution after 72 h treatment in (a) Flp-In^TM^-293, (b) ABCB1/Flp-In^TM^-293, (c) HeLaS3 and (d) KB/VIN, respectively, Figure S3: The apoptosis phenomenon of 72 h treatment in (a) Flp-In^TM^-293, (b) ABCB1/Flp-In^TM^-293 and (c) HeLaS3 cell lines, Table S1: The effects of ZA-A, ZA-B and ZA-C on the transport of rhodamine123 by human P-gp, Table S2: The effects of ZA-A, ZA-B and ZA-C on the transport of doxorubicin by human P-gp, Table S3: The detailed percentage of phases in cell cycle analysis.
