# Supplementary material for: Zhankuic Acids A, B and C from Taiwanofungus camphoratus Act as Cytotoxicity Enhancers by Regulating P-Glycoprotein in Multi-Drug Resistant Cancer Cells
Source: Biomolecules. 2019 Nov 21;9(12):759. doi: 10.3390/biom9120759 (PMC6995581; doi:10.3390/biom9120759)
Supplement: Supplementary file 1 [file biomolecules-09-00759-s001.pdf]

Supplementary materials

Supplementary table 1. The effects of ZA-A, ZA-B, and ZA-C on the transport of rhodamine123 by human P-gp.. 2

Supplementary table 2. The effects of ZA-A, ZA-B, and ZA-C on the transport of doxorubicin by human P-gp.. 3

Supplementary table 3. The detailed percentage of phases in cell cycle analysis.................... 4

Supplementary figure 1. The cytotoxicity of ZA-A, ZA-B, and ZA-C in (a) Flp-In^{TM}-293, (b) *ABCB1*/ Flp-In^{TM}-293, (c) HeLaS3, and (d) KB/VIN, respectively... 6

Supplementary figure 2. The individual results of cell cycle distribution after 72 h treatment in (a) Flp-In^{TM}-293, (b) *ABCB1*/ Flp-In^{TM}-293, (c) HeLaS3, and (d) KB/VIN, respectively........ 9

Supplementary figure 3. The apoptosis phenomenon of 72 h treatment in (a) Flp-In^{TM}-293, (b) *ABCB1*/ Flp-In^{TM}-293, and (c) HeLaS3 cell lines. 13

Supplementary table 1. The effects of ZA-A, ZA-B, and ZA-C on the transport of rhodamine123 by human P-gp.

	Nonlinear kinetic parameters		
ABCB1/Flp-In^{TM}-293	V_m (pmole/10 min)	K_m (μM)	
Rhodamine123 only	13.17 ± 1.64	17.66 ± 3.69	
+ ZA-A, 1 μM	14.09 ± 1.01	39.02 ± 1.38*	
+ ZA-A, 10 μM	13.61 ± 1.81	93.07 ± 0.68*	
K_i			5.54 ± 0.53
Rhodamine123 only	13.37 ± 0.11	22.15 ± 0.48	
+ ZA-B, 1 μM	8.57 ± 0.89*	21.18 ± 4.44	
+ ZA-B, 10 μM	4.98 ± 0.10*	21.50 ± 0.28	
K_i			9.80 ± 1.80
Rhodamine123 only	15.65 ± 1.03	27.07 ± 3.04	
+ ZA-C, 1 μM	15.54 ± 1.02	40.83 ± 1.34*	
+ ZA-C, 10 μM	14.12 ± 0.46	54.27 ± 1.10*	
K_i			11.71 ± 1.73

* $p < 0.05$ as compared to the rhodamine123 transport without ZA-A, ZA-B, or ZA-C.

Supplementary table 2. The effects of ZA-A, ZA-B, and ZA-C on the transport of doxorubicin by human P-gp.

	Nonlinear kinetic parameters		
ABCB1/Flp-In^{TM}-293	V_m (pmole/ 120 min)	K_m (μM)	
Doxorubicin only	12.52 ± 1.12	17.88 ± 2.72	
+ ZA-A, 1 μM	12.02 ± 0.02	38.65 ± 2.44*	
+ ZA-A, 10 μM	12.16 ± 2.85	111.65 ± 0.00*	
K_i			4.68 ± 1.27
Doxorubicin only	30.19 ± 3.04	44.20 ± 2.36	
+ ZA-B, 1 μM	23.09 ± 0.99*	44.97 ± 1.40	
+ ZA-B, 10 μM	17.52 ± 1.43*	45.77 ± 1.99	
K_i			16.40 ± 1.00
Doxorubicin only	19.86 ± 0.55	19.48 ± 0.28	
+ ZA-C, 1 μM	18.11 ± 1.05	33.13 ± 3.33*	
+ ZA-C, 10 μM	17.76 ± 1.36	67.09 ± 3.18*	
K_i			6.81 ± 1.39

* $p < 0.05$ as compared to the doxorubicin transport without ZA-A, ZA-B, or ZA-C.

Supplementary table 3. The detailed percentage of phases in cell cycle analysis.

Flp-In™-293	Percentage of phase ± S.E. (%)			
	**Sub G_1**	**G_0/G_1**	**S**	**G_2/M**
Control	0.6 ± 0.3	48.8 ± 0.2	38.8 ± 0.6	11.8 ± 0.6
Paclitaxel 1 nM	3.3 ± 0.3	41.2 ± 0.2	45.5 ± 0.6	10.0 ± 0.3
ZA-A 20 µM	3.0 ± 0.2	42.7 ± 0.1	45.4 ± 0.1	8.8 ± 0.4
ZA-A 40 µM	3.3 ± 0.1	39.1 ± 0.4	49.3 ± 0.6	8.3 ± 0.2
Paclitaxel 1 nM + ZA-A 20 µM	2.4 ± 0.7	43.0 ± 0.6	45.1 ± 0.8	9.5 ± 0.7
Paclitaxel 1 nM + ZA-A 40 µM	4.2 ± 0.7	40.2 ± 1.0	49.2 ± 0.5	6.5 ± 0.3
***ABCB1*/Flp-In™-293**	Percentage of phase ± S.E. (%)			
	**Sub G_1**	**G_0/G_1**	**S**	**G_2/M**
Control	1.0 ± 0.03	41.5 ± 0.2	45.5 ± 0.3	12.0 ± 0.1
Paclitaxel 250 nM	5.1 ± 1.8	54.6 ± 2.8	13.8 ± 2.6	26.5 ± 0.8
ZA-A 20 µM	1.5 ± 0.04	40.3 ± 0.9	45.5 ± 1.3	12.8 ± 0.5
ZA-A 40 µM	1.3 ± 0.1	37.2 ± 0.4	50.1 ± 0.5	11.4 ± 0.3
Paclitaxel 250 nM + ZA-A 20 µM	14.1 ± 2.0	24.5 ± 0.3	26.2 ± 1.4	35.2 ± 1.5
Paclitaxel 250 nM + ZA-A 40 µM	22.6 ± 0.3	11.8 ± 0.2	27.2 ± 1.3	38.3 ± 1.5
**HeLaS3**	Percentage of phase ± S.E. (%)			
	**Sub G_1**	**G_0/G_1**	**S**	**G_2/M**
Control	0.1 ± 0.02	61.1 ± 0.7	30.9 ± 0.6	8.0 ± 0.2
Paclitaxel 1 nM	2.0 ± 0.6	26.2 ± 1.7	63.0 ± 1.0	8.8 ± 1.2
ZA-A 20 µM	8.3 ± 1.2	52.1 ± 0.5	28.4 ± 0.6	10.9 ± 0.04
ZA-A 40 µM	10.9 ± 0.3	52.0 ± 0.3	28.3 ± 0.9	8.8 ± 0.3
Paclitaxel 1 nM + ZA-A 20 µM	4.8 ± 0.1	27.3 ± 1.4	54.2 ± 0.2	13.8 ± 1.1
Paclitaxel 1 nM + ZA-A 40 µM	2.3 ± 0.3	21.6 ± 0.2	61.3 ± 0.8	14.9 ± 0.5
**KB/VIN**	Percentage of phase ± S.E. (%)			
	**Sub G_1**	**G_0/G_1**	**S**	**G_2/M**
Control	0.3 ± 0.1	57.8 ± 0.2	36.2 ± 0.6	5.8 ± 0.6
Paclitaxel 250 nM	12.9 ± 1.6	53.9 ± 0.2	30.8 ± 1.2	2.4 ± 0.5
ZA-A 20 µM	13.3 ± 2.3	50.6 ± 0.8	32.7 ± 1.2	3.5 ± 0.5
ZA-A 40 µM	10.9 ± 0.6	54.7 ± 0.4	27.7 ± 1.6	6.7 ± 0.5
Paclitaxel 250 nM + ZA-A 20 µM	24.8 ± 1.0	28.2 ± 0.8	12.3 ± 0.3	34.7 ± 0.6
Paclitaxel 250 nM + ZA-A 40 µM	34.3 ± 0.4	20.6 ± 1.6	21.2 ± 2.4	23.8 ± 0.3

Supplementary figure 1.

(a)

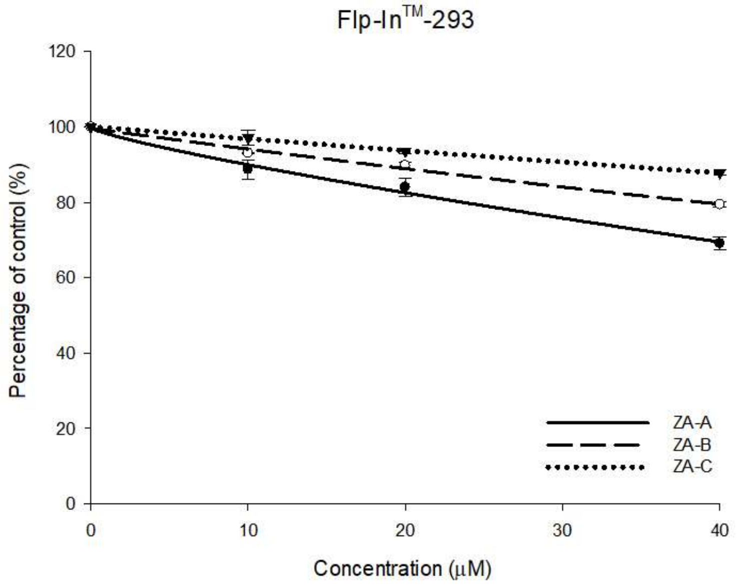

(b)

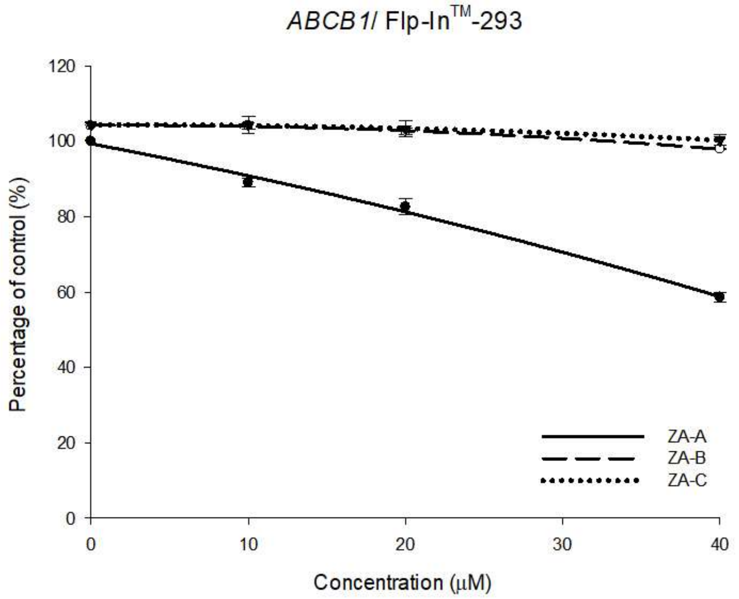

(c)

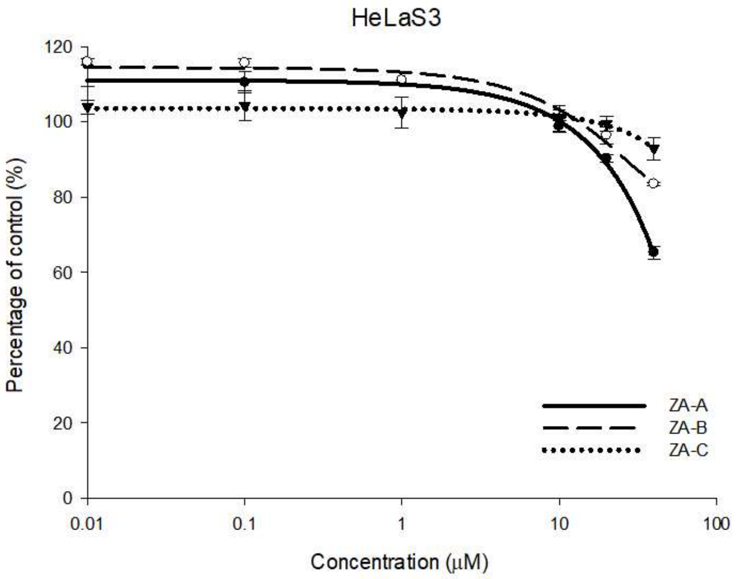

(d)

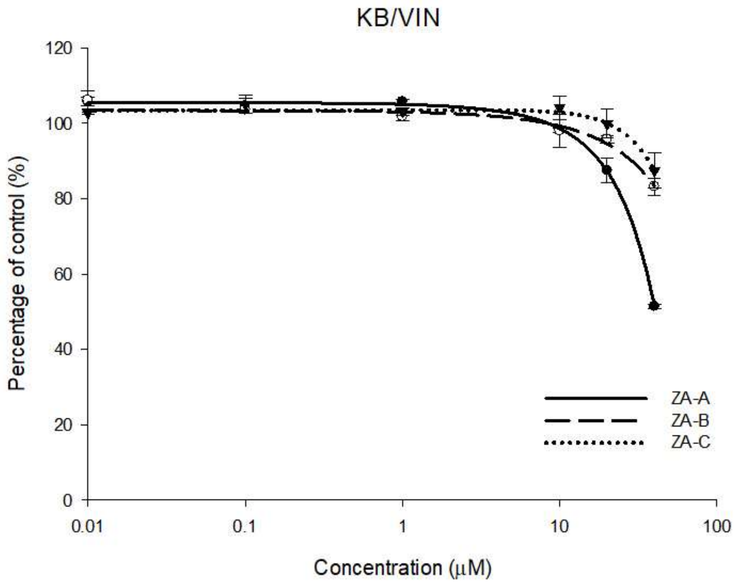

Supplementary figure 1. The cytotoxicity of ZA-A, ZA-B, and ZA-C in (a) Flp-In^{TM}-293, (b) *ABCB1*/ Flp-In^{TM}-293, (c) HeLaS3, and (d) KB/VIN, respectively.

Data were presented as mean ± SE of at least three experiments, each in triplicate.

Supplementary figure 2.

(a)

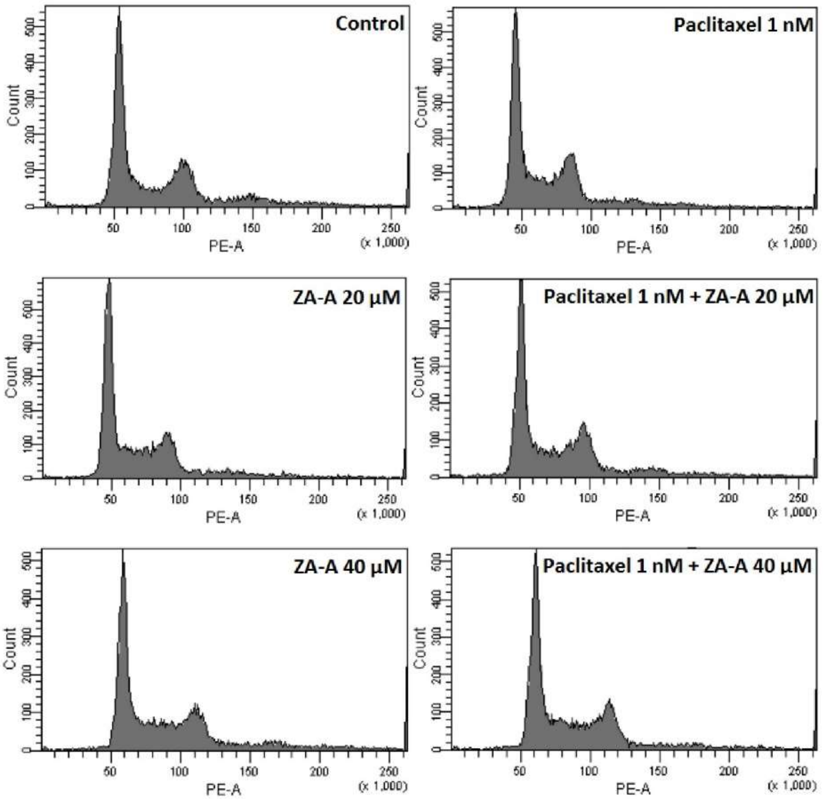

(b)

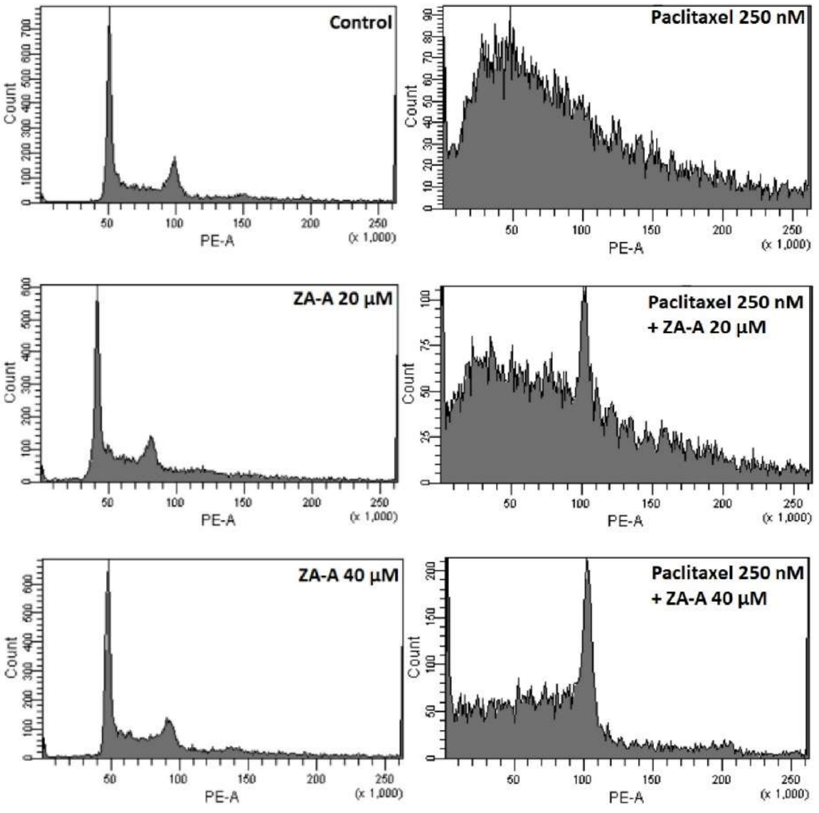

(c)

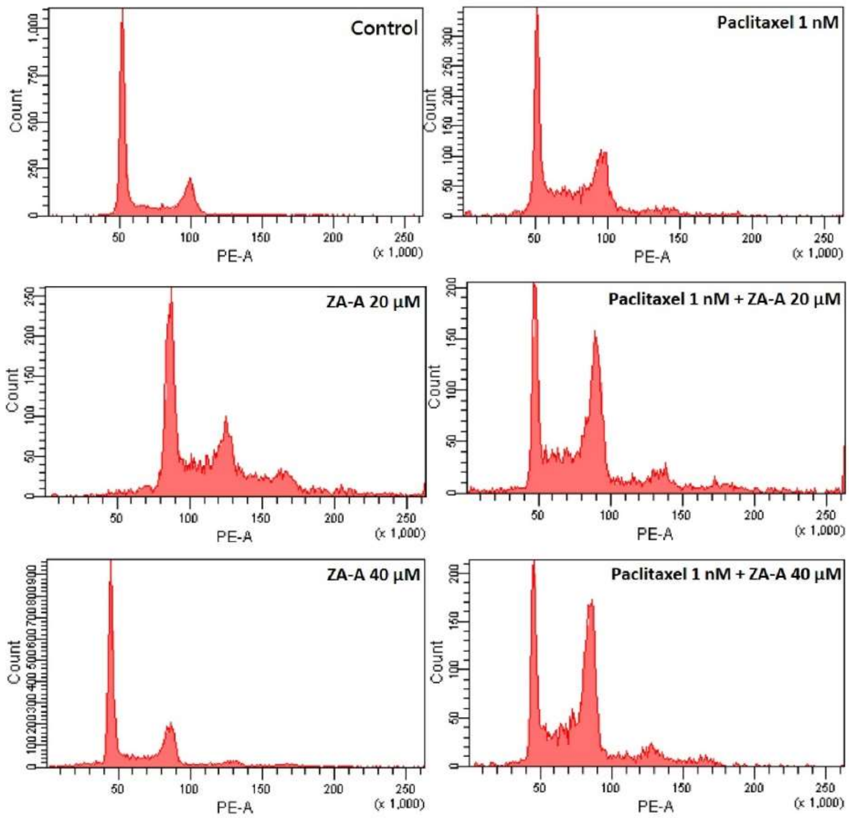
Control
Paclitaxel 1 nM
ZA-A 20 μM
Paclitaxel 1 nM + ZA-A 20 μM
ZA-A 40 μM
Paclitaxel 1 nM + ZA-A 40 μM
Count
PE-A
(x 1,000)

(d)

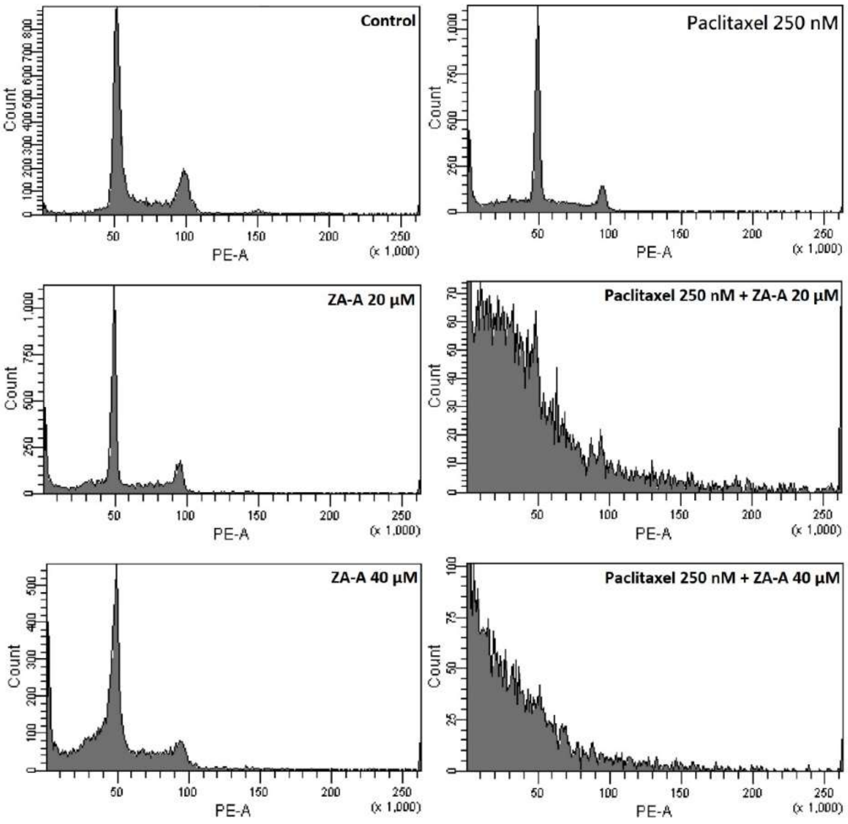
Control
Paclitaxel 250 nM
ZA-A 20 μM
Paclitaxel 250 nM + ZA-A 20 μM
ZA-A 40 μM
Paclitaxel 250 nM + ZA-A 40 μM
Count
PE-A
(x 1,000)

Supplementary figure 2. The individual results of cell cycle distribution after 72 h treatment in (a) Flp-In^{TM}-293, (b) *ABCB1*/ Flp-In^{TM}-293, (c) HeLaS3, and (d) KB/VIN, respectively.

Supplementary figure 3.

(a)

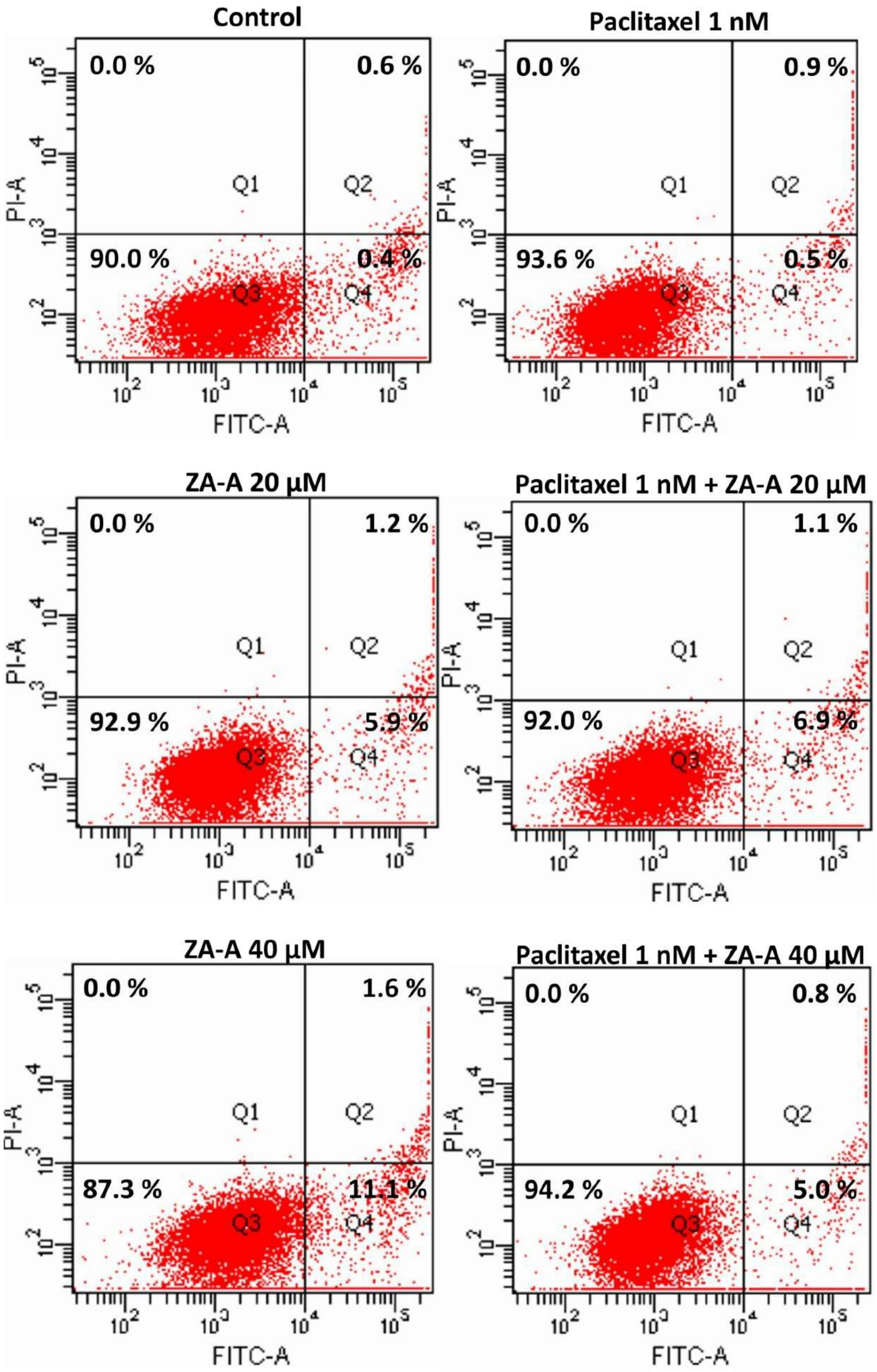

(b)

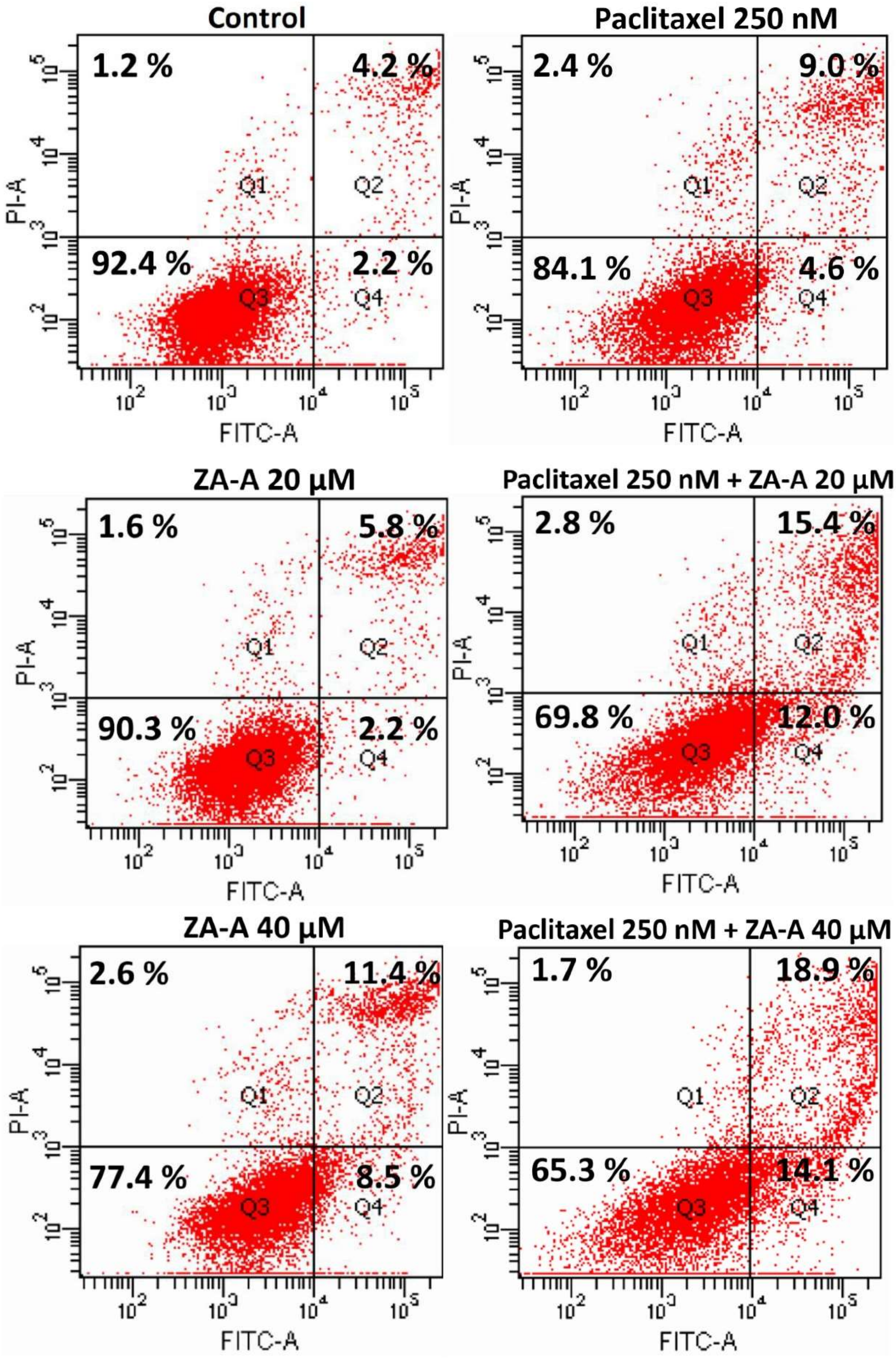

Control
1.2 %
4.2 %
92.4 %
2.2 %
Paclitaxel 250 nM
2.4 %
9.0 %
84.1 %
4.6 %
ZA-A 20 μM
1.6 %
5.8 %
90.3 %
2.2 %
Paclitaxel 250 nM + ZA-A 20 μM
2.8 %
15.4 %
69.8 %
12.0 %
ZA-A 40 μM
2.6 %
11.4 %
77.4 %
8.5 %
Paclitaxel 250 nM + ZA-A 40 μM
1.7 %
18.9 %
65.3 %
14.1 %
Q1
Q2
Q3
Q4
PI-A
FITC-A

(c)

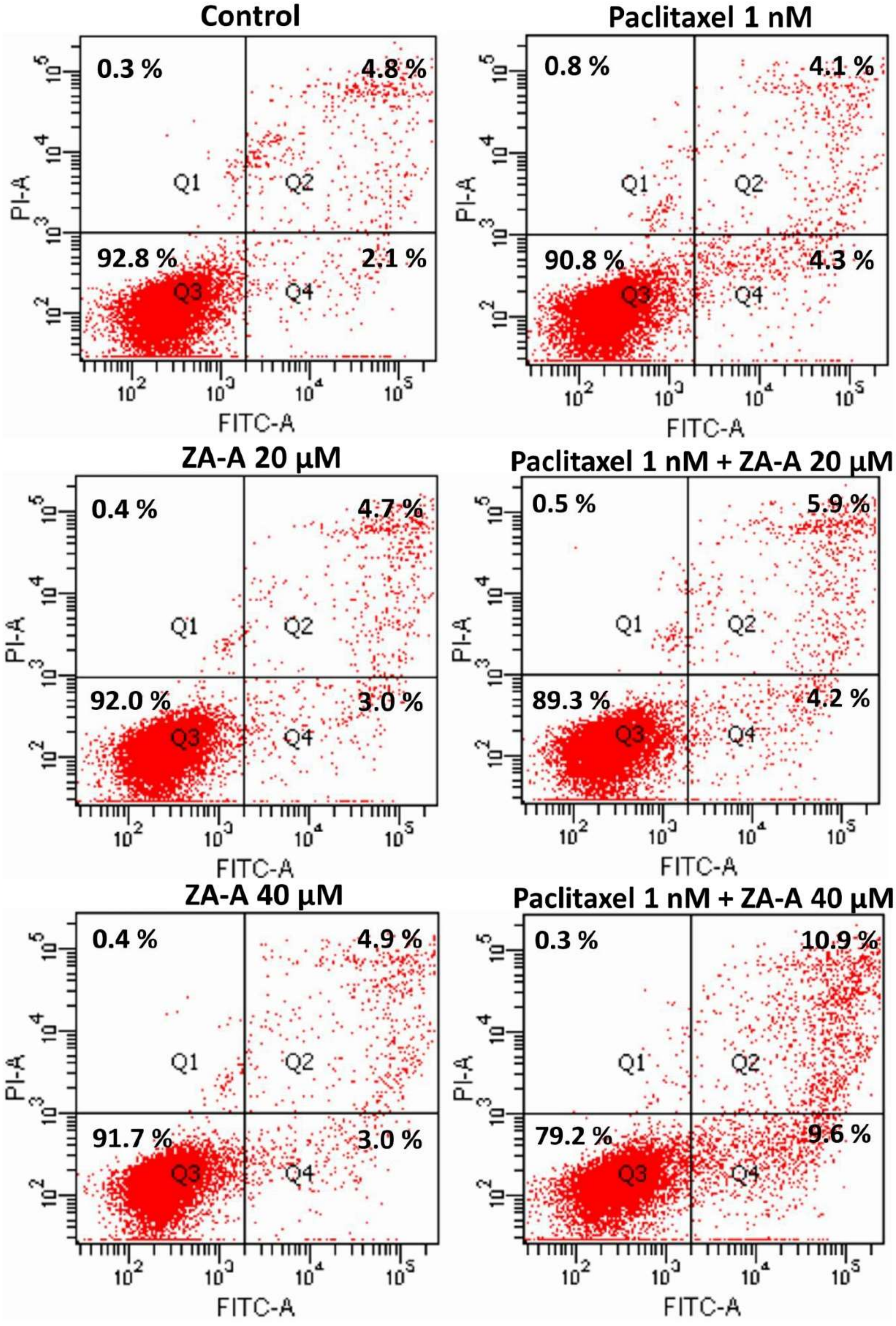

Control
0.3 %
4.8 %
92.8 %
2.1 %
Paclitaxel 1 nM
0.8 %
4.1 %
90.8 %
4.3 %
ZA-A 20 μM
0.4 %
4.7 %
92.0 %
3.0 %
Paclitaxel 1 nM + ZA-A 20 μM
0.5 %
5.9 %
89.3 %
4.2 %
ZA-A 40 μM
0.4 %
4.9 %
91.7 %
3.0 %
Paclitaxel 1 nM + ZA-A 40 μM
0.3 %
10.9 %
79.2 %
9.6 %
Q1
Q2
Q3
Q4
PI-A
FITC-A

Supplementary figure 3. The apoptosis phenomenon of 72 h treatment in (a) Flp-In^{TM}-293, (b) *ABCB1*/ Flp-In^{TM}-293, and (c) HeLaS3 cell lines.

Apoptosis and necrosis status of each sample was determined by annexin V (X-axis FITC) and PI (Y-axis PI). Cell distributed in Q1, Q2, Q3 and Q4 represented necrosis, late-apoptosis, normal and early-apoptosis, respectively.
